# Contribution of cervical incompetence to occurrence of second trimester abortion in patients with polycystic ovary syndrome during the frozen embryo transfer cycle

**DOI:** 10.3389/fendo.2024.1411618

**Published:** 2024-10-16

**Authors:** Tingting Cheng, Hao Shi, Zhiqin Bu, Yiping Yu, Wenyan Song, Jin Haixia, Guidong Yao

**Affiliations:** ^1^ Center for Reproductive Medicine, The First Affiliated Hospital of Zhengzhou University, Zhengzhou, China; ^2^ Henan Key Laboratory of Reproduction and Genetics, The First Affiliated Hospital of Zhengzhou University, Zhengzhou, China

**Keywords:** infertility, cervical incompetence, polycystic ovary syndrome, second-trimester abortion, frozen-embryo transfer

## Abstract

**Background:**

Second-trimester abortion is a critical issue in infertile women with polycystic ovary syndrome (PCOS) treated with assisted reproductive technology (ART), cervical incompetence (CI) may play a role. Although previous studies have revealed an association between PCOS and CI in women undergoing ART with fresh embryo transfer, the associated risk factors in frozen embryo transfer cycles are still unknown. The primary objective of this study is to examine the impact of CI on the occurrence of second-trimester abortion in women with PCOS undergoing frozen embryo transfer.

**Methods:**

This retrospective cohort study included patients who underwent frozen-thawed embryo transfer and experienced second-trimester abortion between January 2012 and January 2020 from the Reproductive Medical Center of the First Affiliated Hospital of Zhengzhou University. Logit-transformed propensity score matching (PSM) was used to assess covariates. Patients were classified into the PCOS and non-PCOS groups. The PCOS group was further divided into two subgroups: the CI group and non-CI group.

**Results:**

After case matching with PSM, 278 patients were included: 139 each in the PCOS group and non-PCOS groups. More miscarriages were attributed to CI in the PCOS group compared with the control group (20.14% vs. 10.07%). Subsequently, in the PCOS group, CI and non-CI subgroup analyses revealed a higher transfer cleavage-stage embryo incidence in the CI group than in the blastocysts group (*p*=0.001). Moreover, the between-group miscarriage-related gestational age varied significantly (*p*=0.039). Binary logistic regression analysis revealed that cleavage embryo transfer (*p*= 0.047) was associated with increased CI risk in the PCOS group, besides, increasing the number of transferred embryos did not impact the occurrence of CI in patients with PCOS.

**Conclusion:**

CI independently predicted a higher risk of second-trimester abortion in patients with PCOS during the frozen embryo transfer cycle. What’s more, increasing the number of transferred embryos did not affect the incidence of CI in the PCOS group.

## Introduction

In women of reproductive age, PCOS is the most prevalent endocrine disorder associated with infertility. It is characterized by hyperandrogenism, anovulation, and polycystic ovaries and associated with a wide range of psychological, reproductive, and metabolic implications affecting lifespan (1, 2). Since patients with PCOS usually experience high levels of circulating androgens as well as insulin resistance, they are expected to have a higher rate of maternal complications during pregnancy, including miscarriage, gestational diabetes, gestational hypertension, and preterm birth (3–5). These complications may occur even after receiving assisted reproductive treatment.

Several studies have highlighted an increased rate of miscarriage in patients with PCOS undergoing ART, particularly in those with high body mass indexes (BMI) or overweight/obesity (6, 7). Approximately 1-2% of pregnancies are anticipated to result in miscarriages during the second trimester (8). However, most studies have focused on outcomes, such as preterm birth rate and live birth rates, gestational diabetes, and gestational hypertension, in patients undergoing fresh embryo transfer cycles. To date, the etiology of second-trimester abortion and its associated risk factors in frozen embryo transfer cycles are largely unknown. Therefore, it is critical and urgent to explore the causes of second-trimester pregnancy loss and swiftly ameliorate them to enhance pregnancy outcomes.

Apart from mid-pregnancy miscarriages caused by PCOS-associated metabolic issues mentioned above, are there maternal physiological factors contributing to poor pregnancy outcomes? Essam have demonstrated that PCOS was characterized by elevated dehydroepiandrosterone (DHEA) and shortened cervical length at three stages of pregnancy (9). Specifically, the cervical length of the PCOS was 22.1mm, which was shorter than 25mm at twenty weeks (10–12), predicting the risk of spontaneous delivery before the due date. These studies make us wonder whether there was a contribution of cervical insufficiency (CI) to the occurrence of second-trimester abortion in patients with PCOS.

CI has received attention as a potential contributing factor to adverse pregnancy outcomes in patients with PCOS. CI is defined as a painless shortening of the cervical canal in the second trimester of pregnancy, accompanied by a dilatation of the cervical os, with or without premature rupture of the membranes, which could lead to protrusion of the amniotic sac out of the cervical os and ultimately result in abortion (13). Patients with PCOS and CI experience a poor prognoses, and the presence of insulin resistance as a comorbidity worsens the prognoses (14). Recent research has indicated a higher frequency of CI in patients with PCOS, particularly in those who receive gonadotropin therapy (15). Similarly, Yang et al. investigated the relationship between CI and PCOS in Chinese women treated using the ART. They found that women with PCOS are more likely to experience CI-complicated pregnancies and have a higher rate of adverse obstetrical outcomes than those without PCOS (16).

This study examined the effects of CI on abortion rates in women with PCOS in their second trimester during the frozen embryo transfer cycle. Moreover, our study serves as an explorative attempt to contribute to the current knowledge about factors that increase the risk of CI in PCOS, and how to protect women who have developed such a condition from an adverse pregnancy outcome.

## Materials and methods

### Study design and patients

Data were provided by the Clinical Reproductive Medicine Management System/Electronic Medical Record Cohort Database (CCRM/EMRCD) of the Reproductive Medical Center of the First Affiliated Hospital of Zhengzhou University for this study. Inclusion criteria were: (1) Patients diagnosed according to the diagnostic criteria established at the 2003 Rotterdam Conference (17, 18); and (2) undergoing a second-trimester abortion at a gestational age between 13 weeks 0 days and 27 weeks 6 days between January 2012 and January 2020. The exclusion criteria were as follows: (1) preimplantation genetic testing cycles; (2) oocyte donation cycles; (3) uterine anomalies: unicornuate/bicornuate/diaphragmatic; (4) cervical conization for cervical lesions (other treatments, such as a loop electrosurgical excision technique (LEEP), cold knife conization, or another cervical surgery performed for gynecological illness indications are also known variables); and (5) age ≥ 40 years.

Given the absence of standardized criteria for the diagnosis of cervical insufficiency, as summarized from the available literatures (19, 20), the diagnostic criteria for CI were as follows: cervical length ≤25 mm with progressive cervical dilatation and shortening of the cervical canal measured by vaginal ultrasound before 24 weeks of gestation, and the amniotic sac protrudes through the cervical opening or into the vaginal canal during the course of this pregnancy termination; or cervical length ≤25 mm measured by vaginal ultrasound when not in gestation, or the passage of a No. 8 cervical dilatation rod through the endocervix without resistance when not in gestation.

### Endometrial preparation

Based on the implantation protocols, all frozen-thawed endometrial preparation cycles were separated into the natural cycle (NC), hormone replacement therapy (HRT), and suppression HRT groups. NC is appropriate for women with regular menstrual cycles. On days 8–10 of menstruation, transvaginal ultrasonography was performed to monitor follicle growth. Follicular and endometrial development parameters were evaluated and combined with estradiol (E2) and luteinizing hormone (LH) levels to determine the ovulation timing. Cleavage embryo or blastocyst transfer was performed on days 3 or 5 of ovulation. Individuals presenting with an irregular menstrual cycle, ovulation dysfunction, or poor endometrial or follicular development were eligible for HRT.

Beginning on days 2–3 of menstruation, 2–4 mg/day of E2 valerate (Progynova, Bayer, Germany) was administered, and both serum E2 levels and transvaginal ultrasound were used to assess endometrial thickness. Suppression of HRT was appropriate for patients with endometriosis stages I-II and endometrial growth restriction in canceled HRT cycles. On the second day of menstruation, 3.75mg of long-acting GnRH-a (Triptorelin Acetate for Injection, IPSEN, France) was administered. After 28 days, serum follicle stimulating hormone (FSH), LH, progesterone (P), and estradiol levels and transvaginal ultrasounds were assessed to confirm the pituitary downregulation effect. The same specifics as those used for the HRT cycles were applied on days 2–5 after withdrawal bleeding. Clinicians decided whether to maintain or increase the original dosage based on the patients’ endometrial thickness. When the endometrial thickness reached a minimum of 7 mm after 12–14 days of treatment, 60 mg of progesterone was administered to decidualize the endometrium. The first progesterone dose was administered before 9 a.m., and the embryo transfer was performed at 10:30 a.m.

### Definition of outcome measures

Demographic data, including age, duration and type of infertility, BMI, basal FSH level, basal LH level, basal testosterone (T) level, endometrial thickness, endometrial preparations, the interval between oocyte retrieval and thawing, number of embryos transferred, embryo developmental stage at transfer, number of miscarriages, and miscarriage gestational weeks were collected from the hospital database and patient files.

What’s more, various abortion factors that comprise CI, fetal factors, maternal factors and other variables. Fetal factors, specifically, included abnormal fetal growth, fetal demise. Maternal factors encompassed gestational hypertension, gestational diabetes mellitus, placental abruption, immune system disorders, vaginal infections, and systemic illnesses (fever, infections, etc.). Other factors encompass maternal exposure to external forces, unexplained factors and subjective refusal to continue the pregnancy. Finally, the primary outcome parameter of the study was the CI rate in the second trimester.

### Statistical analysis

All statistical analyses were performed using SPSS (version 26.0, SPSS Inc., Chicago, USA) software. Continuous variables with a normal distribution are expressed as means and standard deviations. Variables with a non-normal distribution are expressed as medians and interquartile ranges. Student’ t-test or Mann-Whitney U test was used to evaluate differences between the two groups. Chi-square test or Fisher’ s exact test was applied to assess categorical data expressed as frequencies and percentages. Binary logistic regression analysis was performed to examine the independent association between CI and PCOS. Statistical significance was set at *p*< 0.05.

Using the PSM extension in SPSS, a PSM model was developed to reduce bias and enhance comparability between the PCOS and control groups. Age and BMI were used to calculate the propensity scores. A caliper value of 0.01 was specified to ensure accurate matching. Logit-transformed PS matching was performed using a 1:1 ratio protocol without replacement (greedy-matching algorithm). Standardized differences were used to assess the covariate balance. The balance was considered optimal when the standardized difference was <10%.

## Results

### Baseline characteristics of the population

**Figure 1:**
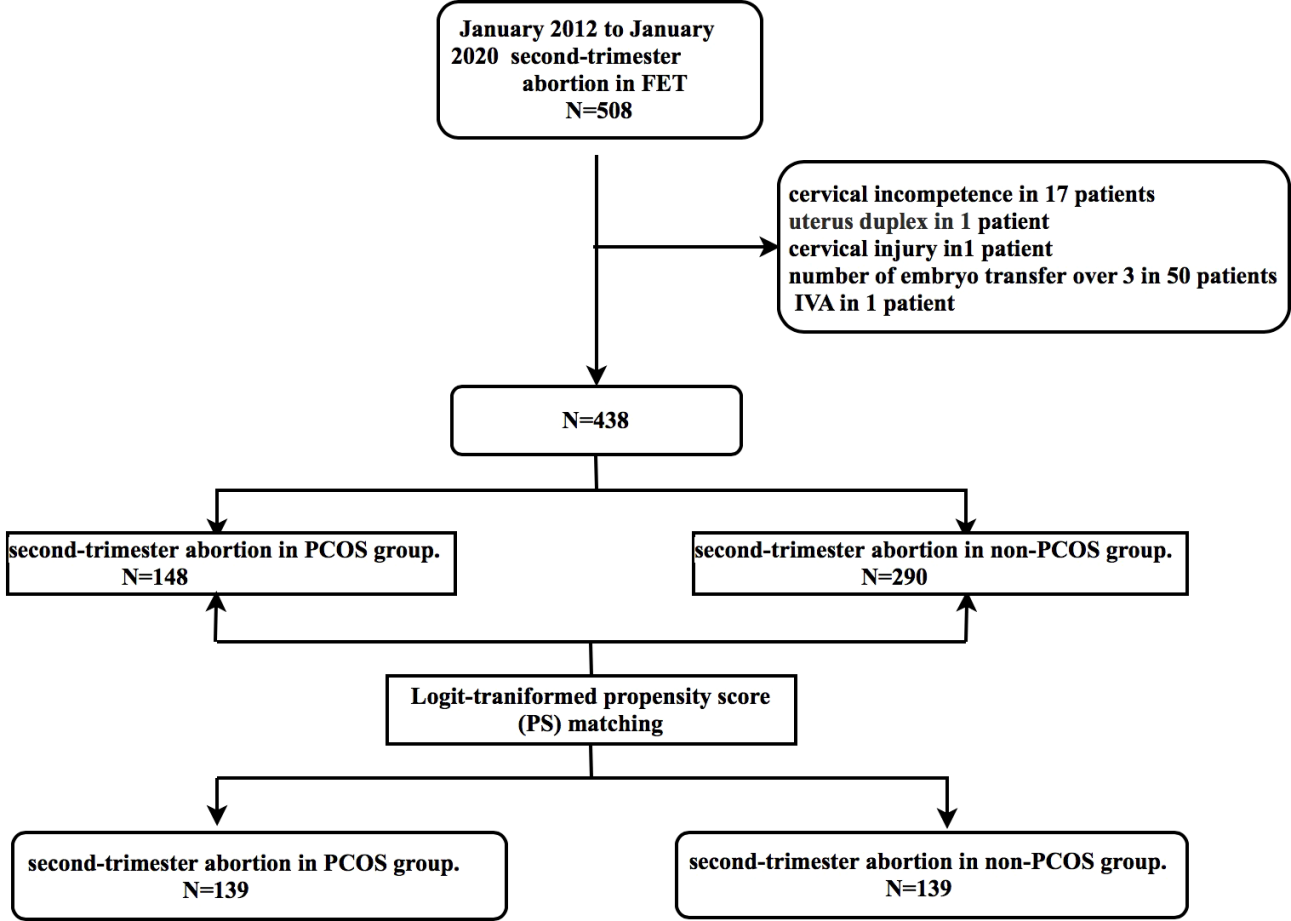
Study flow chart. FET, frozen embryo transfer; IVA, *in vitro* activation; PCOS, polycystic ovary syndrome.

This study eventually included 148 and 290 patients in the PCOS and control groups, respectively. After case matching with PSM, 278 patients were included: 139 each in the PCOS group and non-PCOS groups. Seventy cycles were eliminated from consideration in this study (**Figure 1**). After logit-transformed PSM, 139 patients with PCOS were effectively matched with 139 non-PCOS patients (in the control group) after 1:1 matching. **Table 1** lists the baseline characteristics pre- and post-PSM. Prior to PSM, patients with PCOS were younger (29 vs. 31 years; *p* < 0.001), had a higher BMI (23.95 vs. 22.2 kg/m^2^; *p <*0.001) and basal LH level (8.49 vs. 4.38 mIU/ml; *p* < 0.001), and had a lower level of basal FSH (5.73 vs. 6.10 mIU/ml; *p <*0.001), than did those in the control group. Furthermore, there was a significant between-group difference in infertility type (*p* =0.003). However, no significant between-group differences were found between the two groups in the duration of infertility, endometrial thickness, and the number of embryos transferred. After PSM, no statistically significant between-group differences were observed regarding maternal age, type of infertility, BMI, and basal FSH level. However, basal LH level still showed significant between-group differences.

**Table 1:** Demographic characteristics pre and post-PSM.

	All patients		P	PS-matched	Pairs	P
	PCOS	Non-PCOS		PCOS	Non-PCOS	
Age, years	*29 (26.75-32)*	*31 (28-35)*	<0.001*	*29 (27-33)*	*29 (27-33)*	*0.604*
Duration of infertility Type of infertility Primary Secondary	*4 (2-6)* * * *82 (55.4%)* *66 (44.6%)*	*4 (2-6)* * * *118 (40.7%)* *172 (59.3%)*	*0.413* 0.003*	*4 (2-6)* * * *77 (55.4%)* *62 (44.6%)*	*4 (2-6)* * * *83 (59.7%)* *56 (40.3%)*	*0.642* *0.467*
BMI (Kg/m2)	*23.95 (22.02-26.63)*	*22.22 (20.20-25.39)*	<0.001*	*24.15±3.04*	*24.30±3.25*	*0.277*
Basal FSH (mIU/ml)	*5.73 (4.85-6.81)*	*6.10 (5.22-7.29)*	0.007*	*5.78 (4.88-6.82)*	*5.90 (5.12-7.11)*	*0.238*
Basal LH (mIU/ml)	*8.49 (4.77-14.33)*	*4.38 (3.23-6.02)*	<0.001*	*8.59 (4.78-14.62)*	*4.31 (3.18-5.80)*	<0.001*
Endometrial thickness(mm) No. of embryos transferred	*10 (9-11)* *2 (2-2)*	*10 (9-11)* *2 (2-2)*	*0.104* *0.172*	*10 (9-11)* *2 (2-2)*	*10 (9-11)* *2 (2-2)*	*0.072* *0.144*

BMI, body mass index; FSH, follicle stimulating hormone; LH, luteinizing hormone. Values are mean ± standard deviation; median (Quartile1, Quartile3) or percent (n). *P<0.05, P< 0.05 indicates statistically significant difference between the groups.

### Comparison of the incidence of CI in the PCOS and the non-PCOS groups

**Figure 2:**
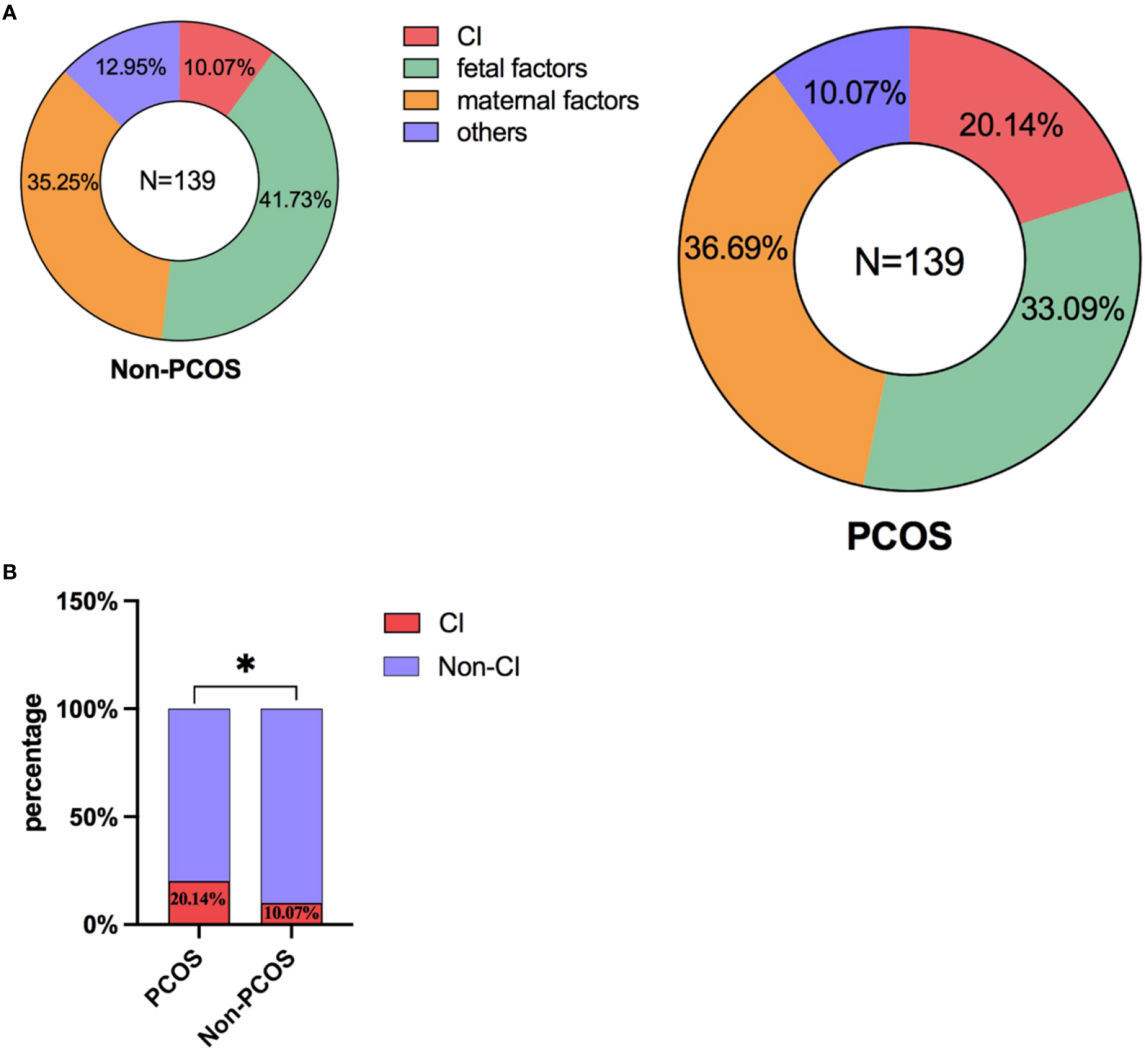
**(A)** The proportion of various abortion factors within both the PCOS and non-PCOS group. **(B)** Comparison of the incidence of CI on the occurrence of second-trimester abortion in the PCOS and non-PCOS groups. *: P<0.05; P<0.05 indicates a statistically significant difference.

As shown in **Figure 2A**, these two pie charts briefly demonstrate the proportion of various abortion factors that comprise CI, fetal factors, maternal factors and other variables within both the PCOS and non-PCOS group. No statistically significant difference was observed between the PCOS group and the non-PCOS group in terms of the percentage of various reasons for second trimester abortion (**Supplementary Table S1**). Notably, CI significantly contributed to second-trimester abortion in the PCOS group compared with that in the non-PCOS group (**Figure 2B**), and the differences in values between the two groups were as follows: 20.14% vs. 10.07%.

### Subgroup analysis in patients with PCOS

Additionally, we performed a subgroup analysis of patients with PCOS who experienced second-trimester abortions with or without CI. Specifically, we found no statistically significant between-group difference in maternal age, BMI, basal FSH levels, serum basic T and the percentage of secondary infertility (**Table 2**). Moreover, we found no effects of the use of the three different endometrial preparation protocols, endometrial thickness and the interval between oocyte retrieval and thawing on the occurrence of CI. There were no significant differences in the number of embryos transferred between the two groups. The difference in the type of embryos transferred between the two groups was significant; thus, there was a higher incidence of transferred cleavage-stage embryos in the CI group than that in the blastocysts group (*p*=0.001), however, since all the women had a miscarriage, we could not conclusively assert that the transfer of blastocysts was more beneficial than that of embryos. The gestational age at which the patients experienced a miscarriage varied considerably between the groups, specifically, that of patients in the CI group was slightly higher than that of women in the control group (22 vs. 20 weeks; *p*=0.039).

**Table 2:** Demographic and clinical characteristics of patients with PCOS with and without CI.

	CI	Non-CI	P
Age (years)	*27 (26-33.5)*	*30 (27-32)*	*0.348*
BMI ( (Kg/m^2^))	*23.52±3.018*	*24.31±3.04*	*0.980*
Basal FSH level Serum basic T	*5.722±1.70* *0.38 (0.22-0.50)*	*5.85±1.32* *0.41 (0.21-0.54)*	*0.081* *0.507*
Type of secondary infertility	*14 (51.9%)*	*46 (41.1%)*	*0.310*
Endometrial preparation			*0.212*
NC group	*2 (7.4%)*	*4 (3.6%)*	
HRT group	*22 (81.5%)*	*81 (72.3%)*	
GnRH-a combined with HRT group	*3 (11.1%)*	*27 (24.1%)*	
Endometrial thickness	*9 (8-10)*	*10 (9-11)*	*0.108*
Miscarriage gestational weeks	*22 (20-23)*	*20 (14-24)*	0.039*
Number of embryos transferred			*0.202*
1	*1 (3.7%)*	*17 (15.2%)*	
2	*26 (96.3%)*	*95 (84.8%)*	
Embryo developmental stage at transfer			<0.001*
Day3	*17 (63%)*	*31 (27.7%)*	
Day5/6	*10 (37%)*	*81 (72.3%)*	
Interval between oocyte retrieval and thawing (month)	*5 (4-12)*	*5 (4-7.75)*	*0.677*

BMI, body mass index; FSH, follicle stimulating hormone; NC, natural cycle; T, testosterone; HRT, the hormone replacement.

therapy; GnRH-a, gonadotropin releasing hormone agonist.

Values are mean ± standard deviation; median (Quartile1, Quartile3) or percent (n*: P<0.05, P< 0.05 indicates statistically significant difference between the groups.).

Regarding the diagnosis of PCOS being limited to the 2003 Rotterdam Criteria, the existence of multiple classification systems caused clinical confusion and hindered scientific progress in comprehending PCOS (21). As a result, in 2012, the NIH organized an evidence-based methodology workshop on PCOS, during which PCOS experts once again suggested utilizing the broader 2003 Rotterdam criteria, while specifically identifying sub-phenotypes within these criteria, including (1) androgen excess and ovulatory dysfunction, (2) androgen excess and PCOM, (3) ovulatory dysfunction and PCOM, and (4) androgen excess, ovulatory dysfunction, and PCOM (22). Based on this subtype classification, we performed a statistical analysis on the PCOS cohort regarding whether or not they exhibited CI (refer to **Supplementary Table S2**). However, given the constraints of our sample size, no significant statistical difference was detected between the groups.

Logistic regression analysis was performed to detect potential confounding factors, including maternal age, BMI, number of miscarriages, serum basal T, number of embryos transferred, embryonic developmental stage at transfer, and gestational week of miscarriage, which were included as independent variables. The embryonic developmental stage at transfer (95% confidence interval: 0.109-0.987; *p*=0.047) and the gestational week of miscarriage (95% confidence interval: 1.038-1.302; *p*=0.009) were both associated with an increased risk of CI (**Table 3**). Most interestingly, increasing the number of transferred embryos did not affect the incidence of CI in the PCOS group.

**Table 3:** Analysis of CI risk factors in women with PCOS undergoing frozen-embryo transfer.

Variables	*OR (95% CI)*	*P*
Age	*1.002 (0.888-1.130)*	*0.979*
BMI	*0.920 (0.786-1.077)*	*0.298*
Number of miscarriages	*0.991 (0.243-4.046)*	*0.990*
Serum basial T	*0.903 (0.527-1.548)*	*0.710*
Number of embryos transferred	*2.332 (0.247-22.005)*	*0.460*
Embryo developmental stage at transfer	*0.328 (0.109-0.987)*	0.047*
Miscarriage gestational week	*1.162 (1.038-1.302)*	0.009*

BMI, body mass index; T, testosterone. *P<0.05; P<0.05 indicates a statistically significant difference.

## Discussion

In this retrospective cohort study, we found that cervical insufficiency could play an important role in the causes of second-trimester abortion in patients with PCOS during the frozen embryo transfer cycle. Moreover, the protocols for endometrial preparation, endometrial thickness, and the number of embryos transferred did not differ significantly. Cervical length monitoring should be paid more attention during the gestational week for evaluating the progression of risk in the future.

Patients with PCOS who undergo assisted reproductive treatment have remarkably more pregnancy complications. Our research was centered on a cohort of individuals with PCOS who had pregnancy loss at the second-trimester stage. Miscarriage during the second trimester remains a considerable concern, and the underlying causes can be multifactorial. Even after conducting the required examinations, the cause of miscarriage during this time may not be known in up to 50% of instances, miscarriages during this time are expected to occur in 1-2% of pregnancies (8, 23), rendering early detection and intervention crucial for managing pregnancy outcomes effectively. Second-trimester abortion has been the subject of extensive study not only because it is one of the most substantial adverse pregnancy outcomes, but also because some of its causes are treatable in advance, allowing the patient to sail through the middle period of her pregnancy. Among the factors investigated, CI has drawn considerable attention among the factors investigated due to its potential impact on second-trimester abortions. Nevertheless, other variables, such as aberrant embryonic development, uterine abnormalities, and external maternal influences, may also play a contributing role. A primary question addressed in the present study was whether or not CI could play a role in patients with PCOS enduring second-trimester abortion in the frozen embryo transfer cycle.

Previous studies have focused on the relationship between PCOS and CI during fresh embryo transfer cycles. Seth et al. suggested that women with PCOS and CI were more likely to have received gonadotropin therapy, although their study was limited by the small number of patients with *in vitro* fertilization (IVF) -conceived pregnancies (15). Furthermore, among patients with CI and PCOS, the cessation of pregnancies at earlier gestational ages and worse pregnancy outcomes have been assessed with and without ART treatment (14). Similarly, in order to investigate the outcomes in specific populations receiving ART treatment, Wu et al. found that the prevalence of CI in women with PCOS undergoing IVF/intracytoplasmic sperm injection (ICSI) treatment was higher than that in those without PCOS by using a nomogram model, indicating the need for close surveillance of cervical changes during the second trimester in these groups (16). However, these studies used fresh embryo transfer cycle data, and little is known about their interaction during the frozen embryo transfer cycle because frozen embryo transfer allows the ovary to recuperate from drug stimulation and the endometrial lining to shed, resulting in a new start for both.

In women with PCOS, transferring frozen embryos results in higher live birth rates and a lower risk of ovarian hyperstimulation than transferring fresh embryos (24). In addition, according to a study by Ali Gökçe et al. (25), hysteroscopic surgery before the ART treatment may increases the risk of CI in the second trimester caused by cervical trauma during mechanical dilatation, which decreases cervical strength during pregnancy, especially in fresh embryo transfer cycles. Despite the limited number of cases, collagen damage is thought to be caused by cervical hyperextension. Consequently, it is essential to investigate the association between CI and PCOS during the thawing cycle.

A previous study has indicated that androgens are essential for cervical remodeling and myometrial maturation (26), which may lead to preterm delivery. However, the study failed to demonstrate a consistent link between androgen excess or androgen deficiency and pregnancy outcomes. By developing a nomogram model to predict the occurrence of CI prior to IVF/ICSI treatment, Wu et al. found that excess androgen levels have a detrimental effect on cervical competence and brought awareness to some high-risk populations of infertile women with risk factors (particularly women with PCOS) (27). However, how androgens affect pregnancy outcomes remains unclear (28). No correlation was found between the incidence of CI and serum testosterone levels in our study. Further studies are required to document the amount of circulating testosterone and the other types of androgen in the mid-trimester pregnancy as to investigate the potential mechanisms of androgen action on cervical remodeling and myometrial contractility.

Another significant finding of the present study was that increasing the number of transferred embryos did not affect the incidence of CI in the PCOS group. Single-embryo transfer is now becoming the dominant approach for reducing the number of multiple pregnancies while maximizing the cumulative live birth rates (29). However, in their analysis of clinical data compiled from ART-assisted conception in patients diagnosed with CI, Jennifer et al. found that one patient underwent a failed fresh embryo transfer. Subsequently, the patient underwent two unsuccessful frozen cycles involving the transfer of a single embryo. With only three embryos remaining, all of which were low quality, it was decided to transfer them. The final favorable outcome of a twin pregnancy with prophylactic cervical cerclage has indeed resulted in a twin delivery, indicating that the optimal number of embryos differs from one individual to another. It is not always possible to transfer one embryo to achieve a favorable pregnancy outcome (30). Therefore, customized transplantation protocols have been developed based on the individual needs of the patient.

Recently, surgical management with cerclage has been established as an effective intervention for patients with CI. Preventive cervical cerclage augments the cervix’s ability to support increased mechanical load, thereby assisting the internal orifice of the cervix in sustaining the weight of the enlarging fetus during the latter stages of pregnancy. This intervention prevents the dilation of the cervical opening, diminishes the potential for ascending infections, and contributes to extending the duration of pregnancy as well as enhancing the survival chances of the neonate, ultimately leading to more favorable pregnancy outcomes (31, 32). What’s more, prophylactic cervical cerclage may enhance reproductive outcomes in infertile patients diagnosed with cervical incompetence who later conceive twin pregnancies through IVF-ET procedures (30). Most recently, a systematic review and meta-analysis of randomized controlled trials revealed that cervical cerclage did not demonstrate a significant impact on the incidence of preterm birth in singleton pregnancies with a short cervical length (≤25 mm) identified after 24 weeks of gestation (33), which prompts us to carefully consider demographic factors, gestational age, and various other elements when selecting cervical cerclage.

What’s more, recent study indicates that endocervical stiffness, assessed through elastography, could potentially contribute to the development of adenomyosis. Adenomyosis negatively impacts female fertility and pregnancy results, particularly relating to preeclampsia, preterm labor, and rates of small-for-gestational age (SGA) infants. We observed a low prevalence of adenomyosis within our study cohort (data not displayed), however, it will be intriguing to observe whether advancements in ultrasound imaging in the future can offer enhanced insights for diagnosing cervical insufficiency and continuously monitoring cervical length throughout pregnancy (34).

In brief, based on the available information, this study represents the most current research comparing CI-associated pregnancy miscarriages in PCOS in the frozen-embryo transfer cycle with subgroup analysis results, making this important for considering the current research focus on pregnancy outcomes. The present study could provide clinicians with additional information to facilitate the development of more individualized treatment plans and pregnancy monitoring guidance for patients with PCOS. Besides, it would be more beneficial for us to obtain more realistic and reliable conclusions using the PSM model.

However, this study has some limitations. First, its retrospective design prevents the full recognition of potential bias factors. However, we used a PSM approach and conducted a subgroup analysis to mitigate confounding effects. Second, owing to the limited number of samples, follow-up studies with large clinical sample sizes are warranted to provide more precise guidance for patients with PCOS during pregnancy.

## Conclusion

In conclusion, this retrospective analysis revealed a significantly higher contribution of CI to second-trimester abortion in patients with PCOS during frozen embryo transfer cycles, especially at 20–23 weeks of gestation. What’s more, increasing the number of transferred embryos did not affect the incidence of CI in the PCOS group. In the future, the potential of utilizing ultrasound imaging or other blood biochemistry techniques for aiding in the early detection of cervical insufficiency and preventive interventions warrants our scrutiny and contemplation; after all, prevention outweighs cure.

## Data Availability

The original contributions presented in the study are included in the article/**Supplementary Material**. Further inquiries can be directed to the corresponding author.
