# Supplementary material for: Contribution of cervical incompetence to occurrence of second trimester abortion in patients with polycystic ovary syndrome during the frozen embryo transfer cycle
Source: Front Endocrinol (Lausanne). 2024 Oct 16;15:1411618. doi: 10.3389/fendo.2024.1411618 (PMC11521832; doi:10.3389/fendo.2024.1411618)
Supplement: Supplementary file 1 [file Table1.docx]

Supplemental Table S1: The proportion of various abortion factors within both the PCOS and non-PCOS group.

|  | PCOS | Non-PCOS | P |
| --- | --- | --- | --- |
| Reasons for second-trimester abortion |  |  | *0.086* |
| CI | 20.14% (28/139) | 10.07% (14/139) |  |
| Fetal factors | 33.09% (46/139) | 41.73% (58/139) |  |
| Maternal factors | 36.69% (51/139) | 35.25% (49/139) |  |
| Others | 10.07% (14/139) | 12.95% (18/139) |  |

PCOS：polycystic ovary syndrome. CI: cervical incompetence.
