# Supplementary material for: Contribution of cervical incompetence to occurrence of second trimester abortion in patients with polycystic ovary syndrome during the frozen embryo transfer cycle
Source: Front Endocrinol (Lausanne). 2024 Oct 16;15:1411618. doi: 10.3389/fendo.2024.1411618 (PMC11521832; doi:10.3389/fendo.2024.1411618)
Supplement: Supplementary file 2 [file Table2.docx]

|  | | CI | Non-CI | P |
| --- | --- | --- | --- | --- |
| sub-phenotypes of PCOS |  | |  | *0.342* |
| androgen excess and ovulatory dysfunction | 11.11% (3/27) | | 8.03% (9/112) |  |
| androgen excess and PCOM | 11.11% (3/27) | | 3.57% (4/112) |  |
| ovulatory dysfunction and PCOM | 55.56% (15/27) | | 58.04% (65/112) |  |
| androgen excess, ovulatory dysfunction and PCOM | 22.22% (6/27) | | 30.36% (34/112) |  |

Supplemental Table S2: Analysis of the proportion of various PCOS subtypes with or without CI among the PCOS cohort.

PCOS：polycystic ovary syndrome. CI: cervical incompetence. PCOM: polycystic-appearing ovarian morphology.
